# Validation of the Italian version of the ANCA-associated vasculitis patient-reported outcome (AAV-PRO) questionnaire

**DOI:** 10.1093/rap/rkae001

**Published:** 2024-01-22

**Authors:** Elena Treppo, Miriam Isola, Maria De Martino, Roberto Padoan, Alessandro Giollo, Maria Letizia Urban, Sara Monti, Silvia Sartorelli, Angelo Fassio, Lorenza Maria Argolini, Chiara Marvisi, Angelica Gattamelata, Francesca Regola, Francesco Ferro, Giulia Cassone, Francesca Motta, Alvise Berti, Edoardo Conticini, Serena Guiducci, Marco Matucci-Cerinic, Alberto Lo Gullo, Andreina Manfredi, Bruno Frediani, Roberto Bortolotti, Carlo Selmi, Chiara Baldini, Franco Franceschini, Fabrizio Conti, Roberto Caporali, Maurizio Rossini, Lorenzo Dagna, Carlomaurizio Montecucco, Giacomo Emmi, Franco Schiavon, Carlo Salvarani, Luca Quartuccio

**Affiliations:** Division of Rheumatology, Department of Medicine, University of Udine, Udine, Italy; Institute of Statistics, Department of Medicine, University of Udine, Udine, Italy; Institute of Statistics, Department of Medicine, University of Udine, Udine, Italy; Rheumatology Unit, Department of Medicine DIMED, University of Padua, Padova, Italy; Rheumatology Unit, Department of Medicine DIMED, University of Padua, Padova, Italy; Department of Experimental and Clinical Medicine, University of Florence, Florence, Italy; Department of Internal Medicine and Therapeutics, Università di Pavia, Pavia, Italy; Division of Rheumatology, Fondazione IRCCS Policlinico San Matteo, Pavia, Italy; IRCCS San Raffaele Scientific Institute, Unit of Immunology, Rheumatology, Allergy and Rare Diseases (UnIRAR), Milano, Italy; Rheumatology Unit, University of Verona, Verona, Italy; Division of Clinical Rheumatology, ASST Istituto Gaetano Pini—CTO, Milano, Italy; Azienda USL-IRCCS di Reggio Emilia and University of Modena and Reggio Emilia, Reggio Emilia, Italy; Department of Clinical Internal, Anesthesiological and Cardiovascular Sciences, Rheumatology Unit, Sapienza University of Rome, Rome, Italy; Rheumatology and Clinical Immunology Unit, ASST Spedali Civili, and Department of Clinical and Experimental Sciences, University of Brescia, Brescia, Italy; Rheumatology Unit, Department of Clinical and Experimental Medicine, University of Pisa, Pisa, Italy; Rheumatology Unit, University of Modena and Reggio Emilia, Modena, Italy; Rheumatology and Clinical Immunology, Humanitas Clinical and Research Center—IRCCS, Rozzano, Milano, Italy; Center for Medical Sciences (CISMed), University of Trento, and Rheumatology Unit, Santa Chiara Hospital, APSS Trento, Italy; Rheumatology Unit, Department of Medicine, Surgery and Neurosciences, University of Siena, Siena, Italy; Rheumatology Unit, Department of Clinical and Experimental Medicine, University of Florence, Italy; Rheumatology Unit, Department of Clinical and Experimental Medicine, University of Florence, Italy; Rheumatology Unit, Garibaldi Hospital, Catania, Italy; Rheumatology Unit, University of Modena and Reggio Emilia, Modena, Italy; Rheumatology Unit, Department of Medicine, Surgery and Neurosciences, University of Siena, Siena, Italy; Center for Medical Sciences (CISMed), University of Trento, and Rheumatology Unit, Santa Chiara Hospital, APSS Trento, Italy; Rheumatology and Clinical Immunology, Humanitas Clinical and Research Center—IRCCS, Rozzano, Milano, Italy; Department of Biomedical Sciences, Humanitas University, Milano, Italy; Rheumatology Unit, Department of Clinical and Experimental Medicine, University of Pisa, Pisa, Italy; Rheumatology and Clinical Immunology Unit, ASST Spedali Civili, and Department of Clinical and Experimental Sciences, University of Brescia, Brescia, Italy; Department of Clinical Internal, Anesthesiological and Cardiovascular Sciences, Rheumatology Unit, Sapienza University of Rome, Rome, Italy; Division of Clinical Rheumatology, ASST Istituto Gaetano Pini—CTO, Milano, Italy; Rheumatology Unit, University of Verona, Verona, Italy; IRCCS San Raffaele Scientific Institute, Unit of Immunology, Rheumatology, Allergy and Rare Diseases (UnIRAR), Milano, Italy; Department of Internal Medicine and Therapeutics, Università di Pavia, Pavia, Italy; Division of Rheumatology, Fondazione IRCCS Policlinico San Matteo, Pavia, Italy; Department of Experimental and Clinical Medicine, University of Florence, Florence, Italy; Centre for Inflammatory Diseases, Monash University Department of Medicine, Monash Medical Centre, Clayton, Victoria, Australia; Rheumatology Unit, Department of Medicine DIMED, University of Padua, Padova, Italy; Azienda USL-IRCCS di Reggio Emilia and University of Modena and Reggio Emilia, Reggio Emilia, Italy; Division of Rheumatology, Department of Medicine, University of Udine, Udine, Italy

**Keywords:** AAV-PRO, ANCA, vasculitis, patient-reported outcome, quality of life, questionnaire, work impairment, glucocorticoids

## Abstract

**Objectives:**

The primary objective of this study was the translation and validation of the ANCA-associated vasculitis patient-reported outcome (AAV-PRO) questionnaire into Italian, denoted as AAV-PRO_ita. The secondary objective was to evaluate the impact of ANCA-associated vasculitis (AAV) on quality of life (QoL) and work impairment in a large cohort of Italian patients.

**Methods:**

The study design took a prospective cohort study approach. First, the AAV-PRO was translated into Italian following the step guidelines for translations. The new AAV-PRO_ita questionnaire covered three disease domains: organ-specific and systemic symptoms and signs; physical function; and social and emotional impact. Second, Italian-speaking AAV patients were recruited from 17 Italian centres belonging to the Italian Vasculitis Study Group. Participants completed the AAV-PRO_ita questionnaire at three time points. Participants were also requested to complete the work productivity and activity impairment: general health questionnaire.

**Results:**

A total of 276 AAV patients (56.5% women) completed the questionnaires. The AAV-PRO_ita questionnaire demonstrated a good internal consistency and test–retest reliability. Female AAV patients scored higher (i.e. worse) in all thee domains, especially in the social and emotional impact domain (*P* < 0.001). Patients on glucocorticoid therapy (*n* = 199) had higher scores in all domains, especially in the physical function domain (*P* < 0.001), compared with patients not on glucocorticoid therapy (*n* = 77). Furthermore, patients who had at least one relapse of disease (*n* = 114) had higher scores compared with those who had never had one (*n* = 161) in any domain (*P* < 0.05). Finally, nearly 30% of the patients reported work impairment.

**Conclusion:**

The AAV-PRO_ita questionnaire is a new 29-item, disease-specific patient-reported outcome measuring tool that can be used in AAV research in the Italian language. Sex, glucocorticoids and relapsing disease showed the greatest impact on QoL.

Key messages

- AAV-PRO_ita questionnaire is a disease-specific patient-reported outcome measuring tool with good internal consistency and test–retest reliability.
- Translated AAV-PRO questionnaires may be included routinely in the clinical evaluation of AAV patients worldwide.
- Sex, glucocorticoids and relapsing disease may influence the quality of life of patients.

## Introduction

ANCA-associated vasculitis (AAV) is a group of systemic disorders involving small-sized blood vessel vasculitis [1, 2]. AAV is a rare disease and encompasses three different entities, namely granulomatosis with polyangiitis (GPA), microscopic polyangiitis (MPA) and eosinophilic granulomatosis with polyangiitis (EGPA).

Considering the improved therapeutic strategies, AAV has evolved mainly from an acute and severe disease to a chronic one, and although the prognosis has improved greatly over the years, patients suffer from the long-term consequences of the disease and its treatment, which, although life-saving, is often associated with significant side effects [3]. Recent papers on AAV have underscored the importance of patient-reported outcomes (PROs) in routine medical evaluation and clinical trials [4]. Given that generic PROs can lack specificity, the OMERACT Vasculitis Working Group identified the need for an AAV-specific PRO to capture the perspective of patients fully. An international steering committee comprising patient partners, methodologists, statisticians and clinicians from the UK, USA and Canada developed and validated a new disease-specific PRO, in line with guidance from the US Food and Drug Administration [5, 6]. The ANCA-associated vasculitis patient-reported outcome (AAV-PRO) questionnaire is the new disease-specific PRO measure for AAV. It is a 29-item disease-specific PRO measure in the English language, proving to be valid, reliable, feasible and able to discriminate among disease states. AAV-PRO could become an extremely useful tool to support physicians in their choice of treatment and to investigate the point of view of patients on disease activity [7].

## Methods

### Objectives

The primary objective of this study was to translate and to assess the internal consistency, feasibility and reliability of the Italian version of the AAV-PRO questionnaire (AAV-PRO_ita).

The secondary purpose was to describe, for the first time, a large cohort of Italian AAV patients, by taking into account three disease domains in the questionnaire {organ-specific and systemic symptoms and signs (SSS); patients’ difficulties in daily life [physical function (PF)]; and social and emotional impact (SEI), including concerns about the future} and by describing the impact of AAV on work productivity/impairment through the simultaneous administration of the work productivity and activity impairment: general health (WPAI: GH) questionnaire.

### Study design

This study had a prospective multicentre observational cohort design. The study complied with established standards for translation, cross-cultural adaptation and validation of questionnaires, following the step guidelines for translations of the *Clinical Outcomes at Oxford University Innovation*. Endorsement from the Faculty of Health and Applied Sciences, University of the West of England, Bristol, UK, University of Bristol School of Clinical Science, Bristol, UK and the University of Oxford, Nuffield Department of Population Health (HSRU), Oxford, UK was obtained.

### Inclusion criteria and data collection

Participants had to have been diagnosed with AAV, be native speakers of Italian, be aged ≥18 years and fulfil the following conditions: confirming that they had AAV; having received either a positive test result for ANCA or a diagnostic biopsy or an angiogram; and currently or previously taking glucocorticoids or one or more other immunosuppressant.

The disease phenotype was defined according to the Chapel Hill Consensus nomenclature, and EGPA patients were also included. ANCA testing was done by standard IIF assay for cANCA and pANCA. PR3 and MPO testing was done by direct ELISA with commercially available kits at the local laboratory. The disease activity was evaluated using the BVAS version 3 (BVASv3). The disease damage was evaluated using vasculitis damage index (VDI). The baseline was defined as time 0, corresponding to the first self-completed 29-item AAV-PRO_ita questionnaire.

Participants were recruited from 17 Italian centres with extensive experience in the diagnosis and treatment of systemic vasculitis, including AAV, and belonging to the Vasculitis Study Group of the Italian Society of Rheumatology. Participants were aware of the nature of the study, its purpose and procedures before they decide to participate.

Each AAV participant self-completed the 29-item AAV-PRO_ita candidate questionnaire during a clinical evaluation (time 0). Five to seven days after they provided baseline responses, participants were sent a repeat 29-item AAV-PRO_ita questionnaire (test–retest). Finally, after 3 months, all participants were again sent the same 29-item AAV-PRO_ita questionnaire. At baseline and after 3 months, AAV participants also self-completed the WPAI: GH questionnaire. The WPAI: GH questionnaire is available here: http://www.reillyassociates.net/WPAIGH__Italian-Italy_.pdf, and it allows researchers to examine the extent of absenteeism, presenteeism and impairment in daily activities [8].

### Statistical analysis

Descriptive statistics included frequency analyses (percentages) for categorical variables and the mean (s.d.), median and interquartile range (IQR) for quantitative variables. Categorical variables were compared using the χ^2^ test or Fisher’s exact test, whereas continuous variables were compared using unpaired Student’s *t*-test or the Mann–Whitney *U*-test for two groups, and one-way ANOVA or the Kruskal–Wallis test for more than two groups, according to the Shapiro–Wilk test establishing whether data were normally or non-normally distributed. Correlations between continuous variables and domain scores were assessed with Spearman’s correlation coefficient (*r*). Regarding missing data, owing to the small percentage of this occurrence, statistics were carried out omitting the cases with missing values. All the analyses were assessed using StataCorp. 2023 (*Stata Statistical Software: Release 18,* StataCorp LLC, College Station, TX).

Additional details regarding the study procedures and sample size calculation are reported in the Supplementary Data S1, available at *Rheumatology Advances in Practice* online.

### Compliance with ethical standards

The study was performed in accordance with the principles of the Declaration of Helsinki and Good Clinical Practice guidelines, and the Department of Medicine Institutional Review Board (IRB) has approved the study (protocol no. 091/2021).

## Results

### Study population

**Figure 1.**
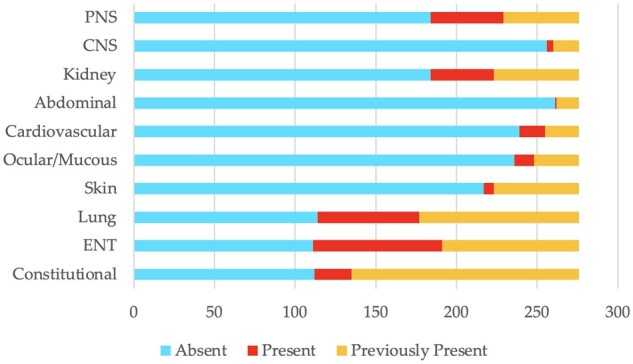
Clinical domains of Italian cohort of ANCA-associated vasculitis patients at baseline *n* = 276. AAV: ANCA-associated vasculitis; CNS: central nervous system; PNS: peripheral nervous system; ENT: ear, nose, throat

A total of 276 participants were enrolled from 17 centres. The median age was 61 (IQR 51.5–71.6) months, and they were predominantly female (156, 56.5%). The types of AAV were GPA (146, 52.9%), EGPA (77, 27.9%) and MPA (53, 19.2%). The ANCA titre was still positive in 108 (39.1%) patients, whereas at disease onset ANCAs were positive in 247 (89.5%) patients [134 of 247 (54.3%) PR3/cANCA and 113 of 247 (45.8%) MPO/pANCA]. The median BVASv3 at baseline was 0 (IQR 0–3), whereas the median BVASv3 at the onset of the disease was 13 (IQR 8–18). Participants had a median illness duration of 62 (IQR 23.8–118.5) months. In their previous medical history, the percentages of patients who experienced at least one relapse, one hospitalization and one severe infection were 41.7, 53.3 and 22.1%, respectively. More than three-quarters of the patients (81.2%) were on immunosuppressant therapy, and 68.8% were still receiving a low dose of glucocorticoids. One hundred and fifty-five of 276 (56.2%) participants belonged to the working-age population, of whom 104 of 155 (67.1%) were in work (Supplementary Fig. S1, available at *Rheumatology Advances in Practice* online). Three of 276 participants died before completing the questionnaire at the third month owing to an infectious disease (two of three with severe acute respiratory syndrome coronavirus 2 infection and one of three with an unknown infection). Demographic and clinical characteristics of participants at baseline are shown in Table 1 and Fig. 1.

**Table 1.** The demographic and clinical characteristics of survey participants at baseline (time 0)

Demographic characteristics	*n* = 276 (%)
Female	156 (56.5)
Male	120 (43.5)
Age, median (IQR), years	61 (51.5–71.6)
Age group	
≤45 years	44 (15.9)
>45, ≤60 years	93 (33.7)
>60, ≤75 years	99 (35.9)
>75 years	40 (14.5)
Ethnicity	
Caucasian	272 (98.5)
Asian	2 (0.7)
African-American	1 (0.4)
Hispanic	1 (0.4)
Type of AAV	
GPA	146 (52.9)
EGPA	77 (27.9)
MPA	53 (19.2)
BVASv3, median (IQR)	0 (0–3)
VDI, median (IQR)	2 (1–4)
On glucocorticoid therapy	199 (72.1)
On immunosuppressant therapy	224 (81.2)
Rituximab (*n* = 224)	73 (32.6)
Methotrexate (*n* = 224)	60 (26.8)
Azathioprine (*n* = 224)	43 (19.2)
Mycophenolate Mofetil (*n* = 224)	23 (10.3)
Cyclophosphamide (*n* = 224)	5 (2.2)
Others (*n* = 224)	20 (8.9)
Time from diagnosis, median (IQR), months	62 (23.8–118.5)
Number of relapses of disease	
0	161 (58.3)
1	69 (25)
≥2	46 (16.7)
Number of hospitalizations	
0	129 (46.7)
1	107 (38.8)
≥2	40 (14.5)
Number of severe infectious	
0	215 (77.9)
1	50 (18.1)
≥2	11 (4)
Working-age patients	155 (56.2)
Employed patients (*n* = 155)	104 (67.1)

AAV: ANCA-associated vasculitis; BVASv3: BVAS version 3; GPA: granulomatosis with polyangiitis; IQR: interquartile range; MPA: microscopic polyangiitis; VDI: vasculitis damage index.

### Measurement properties of AAV-PRO_ita questionnaire

The AAV-PRO_ita questionnaire was self-completed by all 276 participants at baseline, by 268 of 276 (97.1%) participants after 5–7 days, and by 266 of 273 (97.4%) participants at month 3.

**Figure 2.**
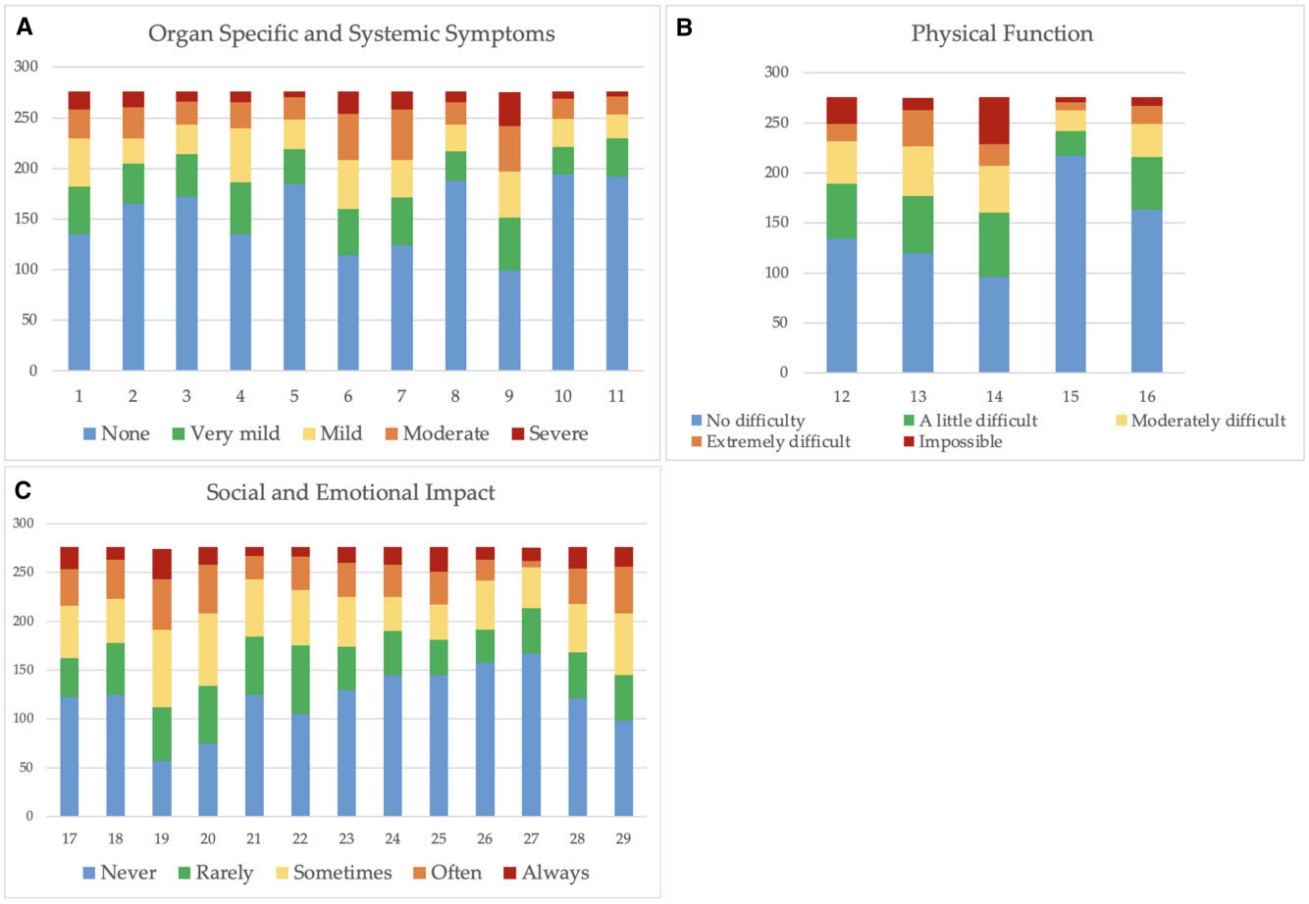
Survey responses at baseline for the 29 items. The *y*-axis shows the number of patients (*n* = 276). The *x*-axis shows the questionnaire items. (**A**) Organ-specific and systemic symptoms and signs. (**B**) Physical function. (**C**) Social and emotional impact

The rate of missing response was 1.8% (432 missing responses out of a total of 23 925 questions). Cronbach’s α ranged from 0.81 to 0.93 (Supplementary Table S1, available at *Rheumatology Advances in Practice* online). The test–retest reliability was 0.95 (95% CI: 0.93, 0.96), 0.94 (95% CI: 0.93 0.95) and 0.95 (95% CI: 0.94, 0.96) in the three disease domains of the questionnaire, respectively (Supplementary Table S2, available at *Rheumatology Advances in Practice* online). The responses to the 29 items at baseline, divided into the three disease domains (SSS, PF and SEI) are shown in Fig. 2.

### Comparison between the AAV-PRO_ita domain scores and demographic and clinical features

There were statistically significant associations between sex, current CS therapy, previous disease relapse and higher scores at baseline. In detail, scores for female patients were higher than those for male patients in all domains (SSS: *P* = 0.047; PF: *P*-value = 0.005; SEI: *P* < 0.001). There were also differences between patients on CS therapy (*n* = 199) and patients without CS therapy (*n* = 77); in fact, the former had higher scores in all domains (SSS: *P* = 0.03; PF: *P* < 0.001; SEI: *P*-value = 0.005). Furthermore, patients who had at least one relapse of the disease (*n* = 114) had higher scores compared with those who had never had one (*n* = 161) (SSS: *P* = 0.013; PF: *P* = 0.015; SEI: *P* = 0.029). No associations were found between CS therapy and previous relapses (*P* = 0.550) or between CS therapy and previous severe infectious (*P* = 0.217).

In contrast, there were no differences in median scores between younger and older responders and across AAV subtypes (GPA, MPA and EGPA). Disease duration, previous hospitalizations and/or infections, BVASv3 and VDI at baseline did not influence the scores. The statistical values for continuous variables are shown in Supplementary Table S3 and the statistical values for non-continuous variables in Supplementary Table S4, both available at *Rheumatology Advances in Practice* online.

### Work productivity and activity impairment: general health questionnaire

Among the 104 working participants, 86 of 104 completed the WPAI: GH questionnaire at baseline and 83 of 104 at the third month. At baseline and at month 3, the percentage of absenteeism was 15% (s.d. 30%) and 8% (s.d. 23%), respectively. In other words, patients were absent from work on average 6 and 3 h per week. The percentage of presenteeism was 22% (s.d. 27%) both at baseline and at month 3. The percentage of overall work impairment was 30% (s.d. 31%) (at baseline) and 27% (s.d. 29%) (at month 3), and the percentage of activity impairment was 36% (s.d. 30%) (both at baseline and at month 3).

Considering the WPAI: GH questionnaire at baseline, the percentage of activity impairment was correlated with a higher score in the PF domain of AAV-PRO_ita [Spearman’s correlation coefficient (*r* > 0.7 is strong correlation), *r* = 0.72, *P* < 0.001] and with the ongoing use of CS therapy (*P* = 0.023).

## Discussion

The AAV-PRO_ita is a new 29-item, disease-specific PRO measure useful in AAV research in the Italian language. This study describes the underlying structure of the final AAV-PRO_ita. It has three disease domains that investigate the impact of symptoms, patients’ difficulties related to physical function and their concerns in daily life. Each domain had good internal consistency (Cronbach’s α ranged from 0.81 to 0.93) and good test–retest reliability (intraclass correlation coefficients ranged from 0.94 to 0.95). Item response rates were high overall (maximum of 1.8% missing data), supporting the feasibility of the questionnaire.

**Figure 3.**
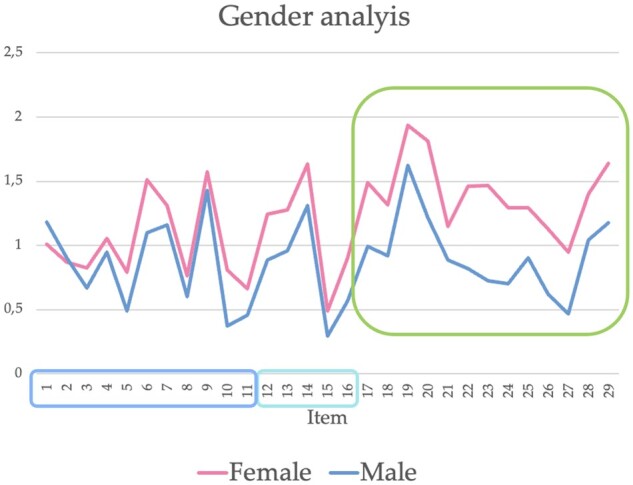
Representation of the mean scores of females and males for each item of the AAV-PRO_ita questionnaire *n* = 276. Light blue frame: organ-specific and systemic symptoms and signs (items 1–11); turquoise frame: physical function (items 12–16); green frame: social and emotional impact (items 17–29)

The female sex scored higher (i.e. worse) in all three domains, especially the SEI domain (*P* < 0.001), in all three stages of questionnaire self-completions (at baseline, after 5–7 days and after 3 months) (Fig. 3). The scores were not influenced by AAV phenotype or disease duration. These results are comparable to those found by Robson *et al.* [6], thus supporting the validity of a sex-based approach in AAV research [9]. Considering that disease activity was similar in our cohort [BVASv3: 0 (IQR 0–3)], the differences observed between sexes might have a clinically meaningful impact. Recognizing the emotional and psychological aspects of living with a chronic disease and promoting open and effective communication between health-care providers and female patients could serve as an example of a sex-based approach. In addition to classical treatment strategies, integrating psychological therapy into the care plan could provide essential emotional support, aid in accepting the chronic nature of the condition and help patients to deal with the unique psychological aspects of their journey. Health-care providers should also stay informed about the research related to sex-specific considerations in chronic disease management. First, not all patients with the same chronic disease will have identical experiences, as exemplified by differences in the perception of chronic pain among men and women; this diversity should be taken into account [10]. Second, sex also contributes to biological differences in innate and adaptative immunity. Recent evidence shows that biologics that stimulate immune function (e.g. vaccines) are generally more efficacious in females than males, and therapies that repress immunity (e.g. checkpoint inhibitors and TNF inhibitors) are more effective in males than females [11].

Current treatment and previous relapses also influenced the results of the questionnaire. Patients on CS therapy and those who experienced at least one relapse of the disease had higher scores in all domains. These findings had not been investigated in the previous validation study of the English questionnaire [6]. Conversely, in the validation study of the English questionnaire [6] and in the preliminary evaluation of our questionnaire [12], older participants (≥65 years old) had higher scores in the PF domain (*P* < 0.05), and younger participants (<65 years old) showed a trend of higher scores in the SEI domain; however, these data were not confirmed by our final results. The VDI and BVASv3 did not appear to be related to the AAV-PRO_ita scores (*r* < 0.3, i.e. weak correlation), suggesting that the perspective of physicians and patients might be very different [7]. This lack of correlation was also confirmed recently by another study [13], encouraging the role of AAV-PRO as an additional and complementary endpoint to traditional clinical instruments (i.e. BVAS and VDI) in clinical trials [14]. It might be assumed that patients who required more CS therapy had a more severe disease; however, both VDI and immunosuppressant therapy were not associated with worse AAV-PRO_ita scores. Given the remission status or low disease activity within the cohort, the doses of glucocorticoids were low, making it impractical to categorize patients based on low, medium or high CS doses. Furthermore, there were no associations between ongoing CS therapy and previous relapses. Adverse effects related to glucocorticoid therapy are also likely to be connected to this finding, further emphasizing the global need for a new CS-sparing approach in AAV.

A recent review [15] highlighted that patients with vasculitis are most affected physically by fatigue, psychologically by anxiety, socially by reduced social participation and financially by functional decline and reduced employment. Furthermore, a recent Mexican study [16] reported that worries about the future scored highest on the Spanish-translated version of the AAV-PRO questionnaire. These findings also emerged in the AAV-PRO_ita questionnaire. Globally, our Italian questionnaire revealed patients’ concerns about general life issues (item 20), the future (item 19) and long-term treatment (item 29). More than half of patients claimed to be sometimes, often or always worried about the future (58.7%; item 19), about the effects of treatment (47.5%; item 29) and being on the whole stressed (51.5%; item 20) at baseline (Fig. 2). Overall, the SEI domain obtained the highest scores, supporting a poorly investigated malaise in AAV patients. As in other systemic autoimmune diseases, the perception of the patient’s QoL is most likely to be influenced by the chronicity of the disease. With new treatments and increased survival, AAV has largely become a chronic condition, with the resulting impact on patients’ daily lives. Concerning the SSS domain, the main symptoms reported by patients were fatigue (item 9), arthralgia (item 6), myalgia (item 7) and dyspnoea (item 1). About one-third of patients classified their respiratory and muscle symptoms as mild, moderate or severe. Approximately 45% and 42% of patients complained of fatigue and arthralgia, respectively. In particular, nearly one-third of patients classified fatigue as moderate or severe, which indicates that it has a significant role in the psychosocial impact of this illness. Currently, there is no specific treatment for fatigue, and fatigue is not addressed in the current management guidelines [17], and this represents a case of unmet needs in AAV [7]. With regard to the PF (i.e. the second domain), one-third of patients found it impossible or extremely difficult to engage in sport or physical activity (item 14). Furthermore, in a sub-analysis, no significant association was observed between higher scores in the PF domain and the presence of residual neuropathic damage (*P* = 0.383). These results highlight the influence and importance of both clinical and bio-psychosocial factors in the perception of life quality.

**Figure 4.**
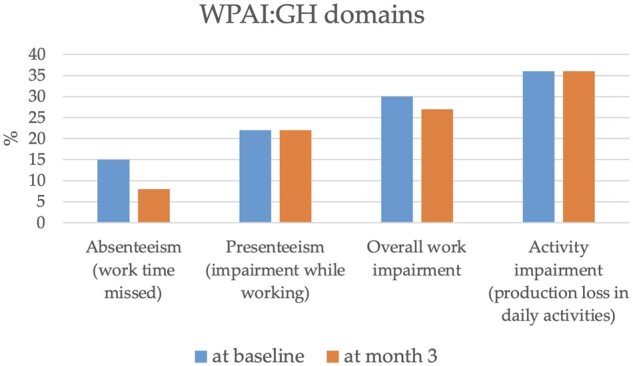
Work productivity and activity impairment (general health) questionnaire in our cohort of ANCA-associated vasculitis patients. WPAI: GH: work productivity and activity impairment (general health)

The impact of work disability is another interesting aspect of AAV deserving further attention. Overall, there were 155 working-age patients, but only 67.1% of them worked. It is likely that AAV might have played a role in this employment rate [18, 19]. A recent Australian study, in which the WPAI: specific health problem questionnaire was administered in 47 AAV patients, reported that ∼25% of responders left paid employment owing to their illness and almost 50% of patients had their financial status impacted [19]. Heron *et al.* [19] found a rate of work disability of 23.4% and observed how work impairment was associated with obesity, lower education and fatigue [20]. Similar to our data, the Australian authors did not find a correlation between BVASv3 and VDI. However, there is no widely accepted definition of work disability; thus, the methods for measuring work disability vary from study to study, making direct comparison between studies and patient groups difficult. Furthermore, to date, few published studies have investigated the impact of work disability in a patient population with systemic vasculitis. In a recent French study, the EXPOVAS study [18], which included 94 patients, the rate of work disability was as high as 40%. An online survey-based study [21] involving 421 North American AAV patients found that 26% of participants became permanently unable to work or had to retire early owing to vasculitis, and the reported mean productivity loss was 7%. In the Australian AAV cohort [19], 10.1% of patients reported missing work in the previous week. This figure is similar to that of our Italian cohort, where absenteeism was ∼8–15% across the two questionnaire completions. The mean activity impairment from our cohort and the Australian cohort was 36% (s.d. 30%) and 26.4% (s.d. 23.6%), respectively. In our study, the percentage of activity impairment seemed to be related to ongoing CS therapy, suggesting once again that CS-sparing approaches could improve AAV management and patients’ QoL. It is also interesting to compare work inability in AAV with that of other rheumatic diseases, such as chronic arthritis. In a previous paper [22], in which the WPAI: GH questionnaire had been administered to a group of Czech patients with arthritis, absenteeism ranged from 8% of patients with RA to 10% of patients with AS, reaching a peak of 20% in patients with PsA owing to the time-consuming treatment and care involved (e.g. phototherapy). Compared with RA and AS, the loss of productivity in our cohort appeared to occur to a lesser extent [22] (Fig. 4). It could be assumed that arthritis influences work productivity more owing to physical impairment. However, neither a DAS in 28 joints [22] nor BVASv3 seemed to be correlated with WPAI: GH. In contrast, the HAQ and the PF domain of the Italian AAV-PRO_ita questionnaire proved to be correlated with work impairment in RA and AAV, respectively. The association between the PF domain of AAV-PRO_ita and work impairment tested by WPAI: GH corroborates the validity of our results. These data suggest an additional evaluation of the impact of the AAV disease on work activity. Further investigation is required to explore this interesting topic and to investigate further the issue of the QoL in AAV patients.

### Strengths and limitations

This study has several strengths. It benefits from a robust sample size, allowing for a comprehensive and statistically meaningful assessment of the performance of the questionnaire. The large number of patients enhances the generalizability of the findings obtained. Additionally, the rigorous methodology adopted in the validation process ensures the reliability and validation of the questionnaire in assessing QoL among AAV patients and helps to clarify which factors might influence it. Furthermore, the absence of an association between the AAV-PRO_ita questionnaire and current clinician instruments, namely VDI and BVAS, supports the idea that this questionnaire complements the overall assessment of AAV patients.

Although the study demonstrates several strengths, it is not without limitations. Potential limitations are the missing data within the cohort and the design of the study. First, missing data arise from non-response by some participants, introducing a bias in the completeness of the analysis. Second, the study might provide a snapshot of the QoL but not capture the dynamic changes that can occur over time. Nevertheless, it is worth noting that the rate of missing response was low, and further studies can deepen the understanding of variations in the perception of life within AAV cohorts over time.

## Conclusions

The AAV-PRO_ita is a new 29-item, disease-specific PRO measure to be used in AAV research in the Italian language. It is a self-administered Italian questionnaire with a good internal consistency, feasibility and reliability. AAV-PRO_ita proved to be a useful tool to explore the perception by AAV patients of their QoL, and it could become an important way of measuring the unmet needs of AAV patients. Nowadays, concerns in daily life seem greatly to influence the health-related QoL of AAV patients, especially in female and working patients. Loss of employment and capacity to work is likely to contribute to loss of one’s status in society, social status and adverse economic consequences for individuals and society at large. These findings also support the validity of research on treatment strategies based on a CS-sparing regimen, showing that patients on a chronic CS therapy had a negative perception of their QoL and working life. Moreover, as with other systemic autoimmune diseases, the problems of fatigue and chronic pain still represent an open challenge for physicians.

This study argues for the need to use validated and user-friendly questionnaires on both QoL and work impairment in order to standardize the results obtained in the different cohorts of AAV patients. In particular, the AAV-PRO questionnaire is easy to use, self-administered, and its translations could be disseminated and included routinely in worldwide clinical evaluation of AAV patients.

## Supplementary Material

rkae001_Supplementary_Data

## Data Availability

The data underlying this article are available in the article and in its online supplementary material.
