# Supplementary material for: Validation of the Italian version of the ANCA-associated vasculitis patient-reported outcome (AAV-PRO) questionnaire
Source: Rheumatol Adv Pract. 2024 Jan 22;8(1):rkae001. doi: 10.1093/rap/rkae001 (PMC10956719; doi:10.1093/rap/rkae001)

## **Supplementary Materials: tables and figures**

**Supplementary Table S1**. Internal consistency of AAV-PRO_ita questionnaire, applying the test Cronbach’s Alpha.

|  | Cronbach’s Alpha | | |
| --- | --- | --- | --- |
| AAV-PRO_Ita | Baseline | After 5-7 Days | After 3 Months |
| Organ Specific And Systemic Symptoms | 0.809 | 0.827 | 0.861 |
| Physical Function | 0.853 | 0.854 | 0.847 |
| Social And Emotional Impact | 0.914 | 0.924 | 0.932 |

AAV, ANCA-associated vasculitis; PRO, patient-reported outcome.

**Supplementary Table S2**. The test-retest reliability, applying the Intraclass correlation coefficients (ICCs).

| AAV-PRO_Ita | ICC | 95% CI |
| --- | --- | --- |
| Organ Specific And Systemic Symptoms | 0.95 | 0.93-0.96 |
| Physical Function | 0.94 | 0.93-0.95 |
| Social And Emotional Impact | 0.95 | 0.94-0.96 |

AAV, ANCA-associated vasculitis; PRO, patient-reported outcome; ICC, intraclass correlation coefficient; CI, confidence interval.

**Supplementary Table S3**. Correlations between continuous variables and domain scores. Spearman’s correlation coefficient (r) was used.

|  |  |  | Spearman’s coefficient (r) | p-value |
| --- | --- | --- | --- | --- |
| VDI | Baseline | SSS | 0.254 | <0.001 |
|  |  | PF | 0.214 | <0.001 |
|  |  | SEI | 0.206 | <0.001 |
|  | After 5-7 Days | SSS | 0.226 | <0.001 |
|  |  | PF | 0.196 | 0.001 |
|  |  | SEI | 0.238 | <0.001 |
|  | After 3 Months | SSS | 0.234 | <0.001 |
|  |  | PF | 0.206 | <0.001 |
|  |  | SEI | 0.187 | 0.002 |
| BVASv3 | Baseline | SSS | 0.178 | 0.003 |
|  |  | PF | 0.304 | <0.001 |
|  |  | SEI | 0.095 | 0.117 |
|  | After 5-7 Days | SSS | 0.152 | 0.014 |
|  |  | PF | 0.269 | <0.001 |
|  |  | SEI | 0.117 | 0.057 |
|  | After 3 Months | SSS | 0.060 | 0.336 |
|  |  | PF | 0.258 | <0.001 |
|  |  | SEI | 0.107 | 0.082 |
| Duration of disease | Baseline | SSS | 0.137 | 0.024 |
|  |  | PF | 0.109 | 0.074 |
|  |  | SEI | 0.086 | 0.159 |
|  | After 5-7 Days | SSS | 0.114 | 0.065 |
|  |  | PF | 0.068 | 0.273 |
|  |  | SEI | 0.082 | 0.186 |
|  | After 3 Months | SSS | 0.138 | 0.025 |
|  |  | PF | 0.094 | 0.129 |
|  |  | SEI | 0.090 | 0.147 |

SSS, organ specific and systemic symptoms and signs; PF, difficulties of the patient in daily life (physical function); SEI, social and emotional impact.

**Supplementary Table S4**. Associations between non-continuous variables and domain scores. T-test or Mann-Whitney U test were used in the case of two groups, ANOVA or Kruskal-Wallis test were used in the case of three groups.

| Sex | | | | |  |
| --- | --- | --- | --- | --- | --- |
| Baseline |  | Males (N=120) | Females (N=156) | **p-value** |  |
|  | SSS, median IQR) | 7 (3.5-14) | 10 (5-16) | **0.047** |  |
|  | PF, median (IQR) | 3 (0-6) | 4 (2-9) | **0.005** |  |
|  | SEI,median (IQR) | 10 (5-16) | 17 (9-26) | **<0.001** |  |
| After 5-7 Days |  |  |  |  |  |
|  | SSS, median IQR) | 7 (3-14) | 9.5 (5-16) | 0.081 |  |
|  | PF, median (IQR) | 2 (0-6) | 4 (2-9) | **0.008** |  |
|  | SEI,median(IQR) | 9 (4-16) | 17 (8-27.5) | **<0.001** |  |
| After 3 Months | |  |  |  |  |
|  | SSS, median IQR) | 7 (4-13) | 9 (5-16) | **0.042** |  |
|  | PF, median (IQR) | 2 (0-6) | 4 (1-8) | **0.024** |  |
|  | SEI, median (IQR) | 8 (4-15) | 17 (9-26) | **<0.001** |  |
| Steroid therapy | | | | |  |
| Baseline |  | Without steroid therapy (N=77) | On steroid therapy  (N=199) | **p-value** |  |
|  | SSS, median (IQR) | 7 (4-12) | 10 (5-16) | **0.030** |  |
|  | PF, median (IQR) | 2 (0-5) | 4 (1-9) | **<0.001** |  |
|  | SEI, median (IQR) | 10 (4-18) | 15 (8-23) | **0.005** |  |
| After 5-7 Days |  |  |  |  |  |
|  | SSS, median (IQR) | 7 (4-12) | 10 (4-16) | **0.044** |  |
|  | PF, median (IQR) | 2 (0-5) | 4 (2-9) | **0.004** |  |
|  | SEI, median (IQR) | 9.5 (4-22) | 15 (7-23) | **0.026** |  |
| After 3 Months | |  |  |  |  |
|  | SSS, median (IQR) | 7 (4-13) | 9 (4-15) | 0.156 |  |
|  | PF, median (IQR) | 2 (1-5) | 4 (1-8) | **0.042** |  |
|  | SEI, median (IQR) | 10 (4-21) | 14 (6-24) | **0.021** |  |
| Disease relapse | | | | | |
| Baseline |  | No relapse (N=161) | At least one relapse (N=114) | **p-value** |  |
|  | SSS, median (IQR) | 7 (4-14) | 11 (5-16) | **0.013** |  |
|  | PF, median (IQR) | 3 (1-6) | 5 (2-8) | **0.015** |  |
|  | SEI, median (IQR) | 11 (5-22) | 15.5 (8-23) | **0.029** |  |
| After 5-7 Days | |  |  |  |  |
|  | SSS, median (IQR) | 8 (4-14) | 10 (5-17) | 0.065 |  |
|  | PF, median (IQR) | 3 (1-7) | 5 (2-8) | **0.027** |  |
|  | SEI, median (IQR) | 12 (5-21.5) | 16 (8-24) | **0.019** |  |
| After 3 Months | |  |  |  |  |
|  | SSS, median (IQR) | 7 (4-14) | 9 (5-17.5) | 0.126 |  |
|  | PF, median (IQR) | 3 (1-7) | 4 (1-8) | **0.047** |  |
|  | SEI, median (IQR) | 10 (4-21) | 14 (6-24) | **0.021** |  |
| Age | | | | |  |
| Baseline |  | <65 years (N=165) | ≥65 years (N=111) | **p-value** |  |
|  | SSS, median (IQR) | 8 (4-14) | 10 (5-16) | 0.251 |  |
|  | PF, median (IQR) | 3 (1-7) | 4 (1-9) | 0.059 |  |
|  | SEI, median (IQR) | 15 (7-23) | 11 (5-23) | 0.123 |  |
| After 5-7 Days | |  |  |  |  |
|  | SSS, median (IQR) | 9 (4-15) | 9 (4-15) | 0.727 |  |
|  | PF, median (IQR) | 3 (1-7) | 4 (2-8) | 0.126 |  |
|  | SEI, median (IQR) | 15 (6-25) | 12 (6-21) | 0.108 |  |
| After 3 Months | |  |  |  |  |
|  | SSS, median (IQR) | 8 (4-14) | 8 (5-14) | 0.642 |  |
|  | PF, median (IQR) | 3 (1-7) | 4 (2-8) | 0.130 |  |
|  | SEI, median (IQR) | 13 (6-23) | 12 (4-21) | 0.172 |  |
|  |  | **AAV subtypes** |  |  |  |
| Baseline |  | EGPA (N=77) | GPA (N=146) | MPA (N=53) | **p-value** |
|  | SSS, median (IQR) | 10 (5-15) | 8 (4-14) | 9 (5-15) | 0.374 |
|  | PF, median (IQR) | 3 (1-9) | 3 (1-6) | 5 (2-8) | 0.172 |
|  | SEI, median (IQR) | 14 (6-23) | 12 (6-20) | 16 (9-25) | 0.185 |
| After 5-7 Days | |  |  |  |  |
|  | SSS, median (IQR) | 10 (5-16) | 8 (4-14) | 9.5 (4-14) | 0.322 |
|  | PF, median (IQR) | 4 (1-9) | 3 (1-6) | 5 (2-9) | 0.133 |
|  | SEI, median (IQR) | 15 (6-24) | 12 (6-20) | 16 (8-26) | 0.202 |
| After 3 Months | |  |  |  |  |
|  | SSS, median (IQR) | 9 (5-15) | 4 (7.5-13) | 9 (4-14) | 0.369 |
|  | PF, median (IQR) | 4 (1-7) | 3 (1-7) | 4.5 (2-9) | 0.111 |
|  | SEI, median (IQR) | 11 (5-23.5) | 11 (4-22) | 16 (10-31) | **0.044** |

Legend: SSS, organ specific and systemic symptoms and signs; PF, difficulties of the patient in daily life (physical function); SEI, social and emotional impact; IQR, inter-quartile range; MPA, microscopic polyangiitis; GPA, granulomatosis with polyangiitis; EGPA, eosinophilic granulomatosis with polyangiitis.

**Supplementary Figure S1**. Representation of employment status of Italian cohort of AAV patients (n=276).


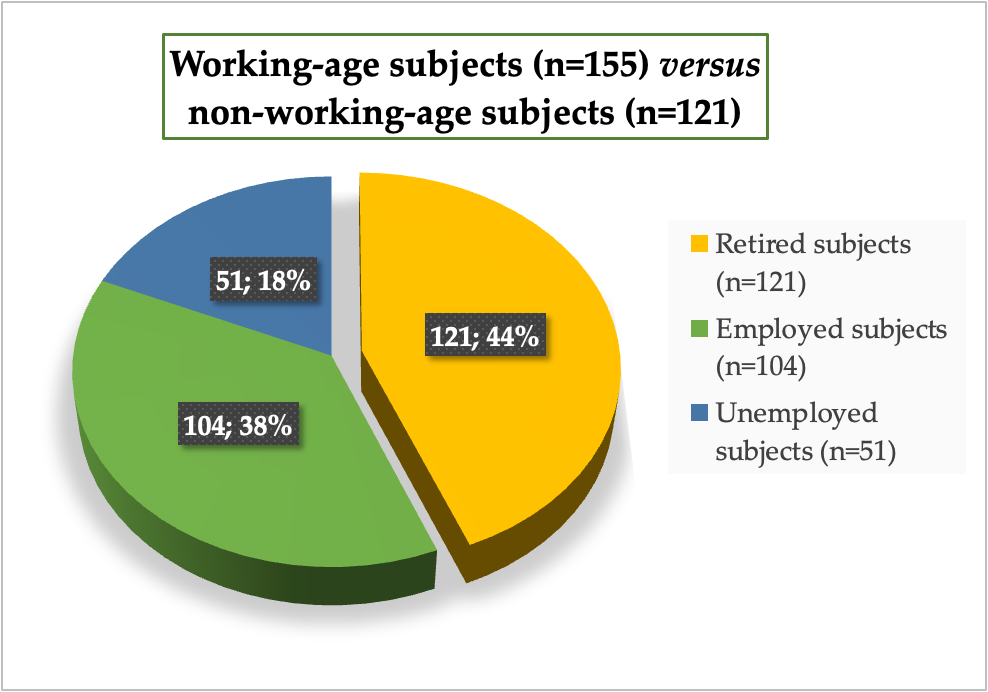

Supplement: rkae001_Supplementary_Data [file rkae001_supplementary_data.zip › 23-085 Supplementary material.docx]
