# Supplementary material for: Validation of the Italian version of the ANCA-associated vasculitis patient-reported outcome (AAV-PRO) questionnaire
Source: Rheumatol Adv Pract. 2024 Jan 22;8(1):rkae001. doi: 10.1093/rap/rkae001 (PMC10956719; doi:10.1093/rap/rkae001)

**Supplementary Data S1. Supplementary Materials: Methodological approach**

### *Study duration*

This is a four-year study that comprises:

- Enrolment phase, including the translation phase: 10 months (from January 2019 to October 2019)
- Data collection: 36 months (from September 2019 to September 2022)
- Follow-up: 3 months
- Analysis: 3 months (from October 2022 to December 2022)

As this is a cohort study, the STROBE statement checklist was followed. The STROBE flow diagram is located at the end of the Supplementary Materials.

###

### *Study procedures*

### *Translation phase*

The translation process followed the principles of simplicity and terminological comprehensibility. The translation from English into Italian was performed by two experts in the English language in close collaboration with the Authors. The questionnaire was then independently back-translated into English by two native speaker translators who had not participated in the previous phases. The two versions were compared and examined by the Authors, who then proceeded to the elaboration of the final version in the Italian language.

#### Pre-test phase (cross-cultural adaptation)

The revised version of the AAV-PRO_ita was administered to volunteer subjects in order to verify its comprehensibility. The volunteer subjects were 2 AAV patients belonging to the Centre promoting the project (i.e., Rheumatology Unit of Udine), that filled the AAV-PRO_ita questionnaire together with their Rheumatologist.

### *Final structure of the candidate questionnaire*

The final version of the AAV-PRO_ita questionnaire had 29 items, divided in three domains of interest: (1) organ specific and systemic symptoms and signs (SSS) [11 items], (2) patients’ difficulties in daily life (physical function [PF]) [5 items], (3) social and emotional impact (SEI), including concerns about the future [13 items]. Each item had five response options, scored from 0 (no problem, never) to 4 (impossible, always). Highest scores denoted a greater severity of impact. The scores for each domain were calculated as the sum of the scores for each individual item. The final version of AAV-PRO_ita is enclosed at the end of document.

### *Verification of the validity and reliability phase*

The statistical evaluation of internal consistency was carried out by applying the Cronbach’s Alpha test. The intra-rater reliability was assessed through the estimation of intraclass correlation coefficients (ICCs), comparing baseline AAV-PRO_ita domain scores, with scores obtained 5-7 days later in those individuals whose condition had remained stable.

1. *Clinical assessments and timelines*

Patients were recruited between September 2019 and September 2022. The Italian Centres involved were the Rheumatology Unit and/or Immunology Unit of (in alphabetical order): Brescia, Catania, Florence, Milan, Modena, Padua, Pavia, Pisa, Reggio Emilia, Rome, Siena, Trento, Udine, Verona. The data were analysed between October and December 2022. Each AAV-participant self-completed the 29-item AAV-PRO_ita candidate questionnaire during a clinical evaluation, i.e. at baseline (t0). Five to seven days after they provided baseline responses, participants were sent a repeat 29-item AAV-PRO_ita questionnaire (test–retest). Finally, after 3 months, all participants were sent again the same 29-item AAV-PRO_ita questionnaire. Demographic (date of birth, location, gender, race) and disease-related data (type of AAV, date of diagnosis, positive ANCA test, current disease state, immunosuppressant medications) were collected by investigators. At baseline and after 3 months, AAV-participants self-completed also the Work Productivity and Activity Impairment (general health) (WPAI:GH) questionnaire. A working person was defined as an individual who is actively engaged in employment or a job, typically for the purpose of earning a living, generating income, or contributing to the workforce. The WPAI:GH questionnaire is available here: *http://www.reillyassociates.net/WPAI-GH__Italian-Italy_.pdf,* and allows researchers to examine the extent of absenteeism, presenteeism, and impairment in daily activities. The WPAI-GH has six questions (Q): current employment status (Q1), number of hours missed due to health problem (Q2), number of hours missed due to other reasons (Q3), hours actually worked (Q4), degree to which health affected productivity while working (Q5), degree to which health affected regular (nonwork) activities (Q6). Four outcome (OC) scores can be derived from the WPAI:GH: OC1, the percentage of work time missed due to health [Q2/(Q2+Q4), percentage of absenteeism], OC2, the percentage of impairment while working due to health [Q5/10, percentage of presenteeism], OC3, the percentage of overall work impairment due to health [Q2/(Q2+Q4)+[(1-Q2/(Q2+Q4))x(Q5/10)]], OC4, the percentage of activity impairment due to health [Q6/10].

### *Sample size*

The sample size for the health status questionnaire development required at least three respondents per questionnaire item tested. The aim was to recruit at least 265 patients. A sample size of 265 patients produces a two-sided 95% confidence interval with a precision of 6.5% when the estimate of Pearson's product-moment correlation is 0.70 (construct validity). This sample size of 265 patients who are each measured 3 times produce a two-sided 95% confidence interval (CI) with precision of 2% when the estimated intraclass correlation is 0.90 (test–retest).


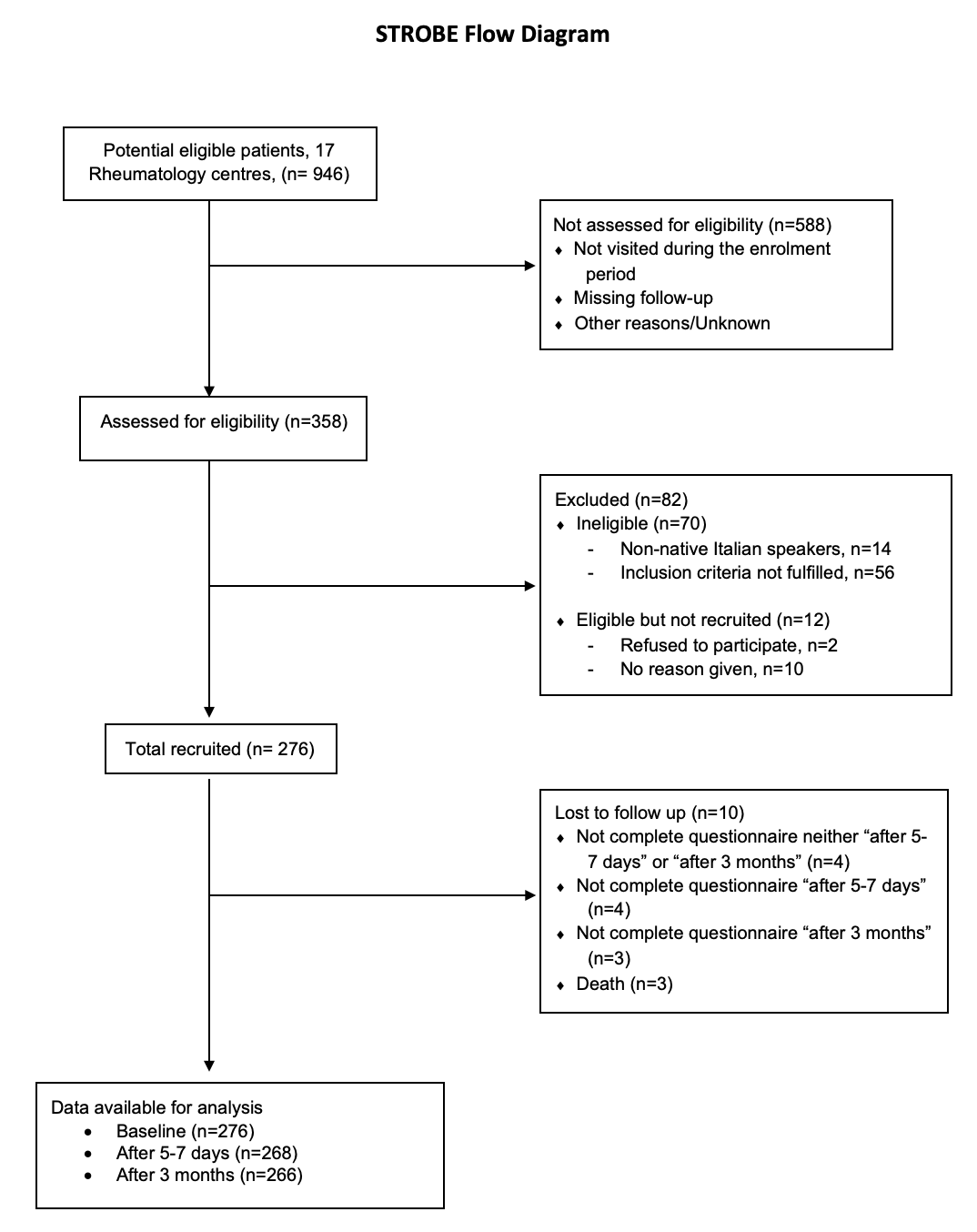


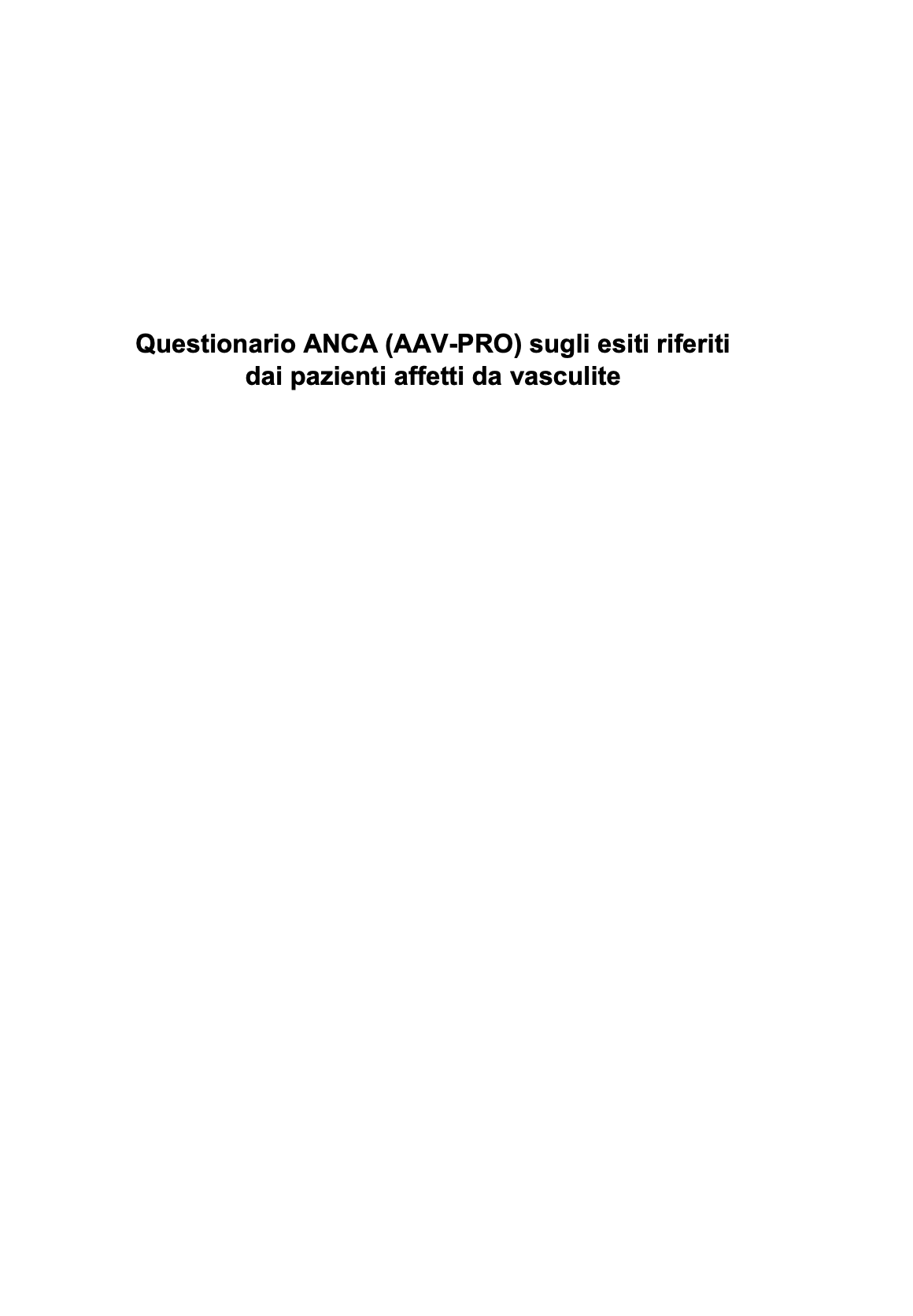


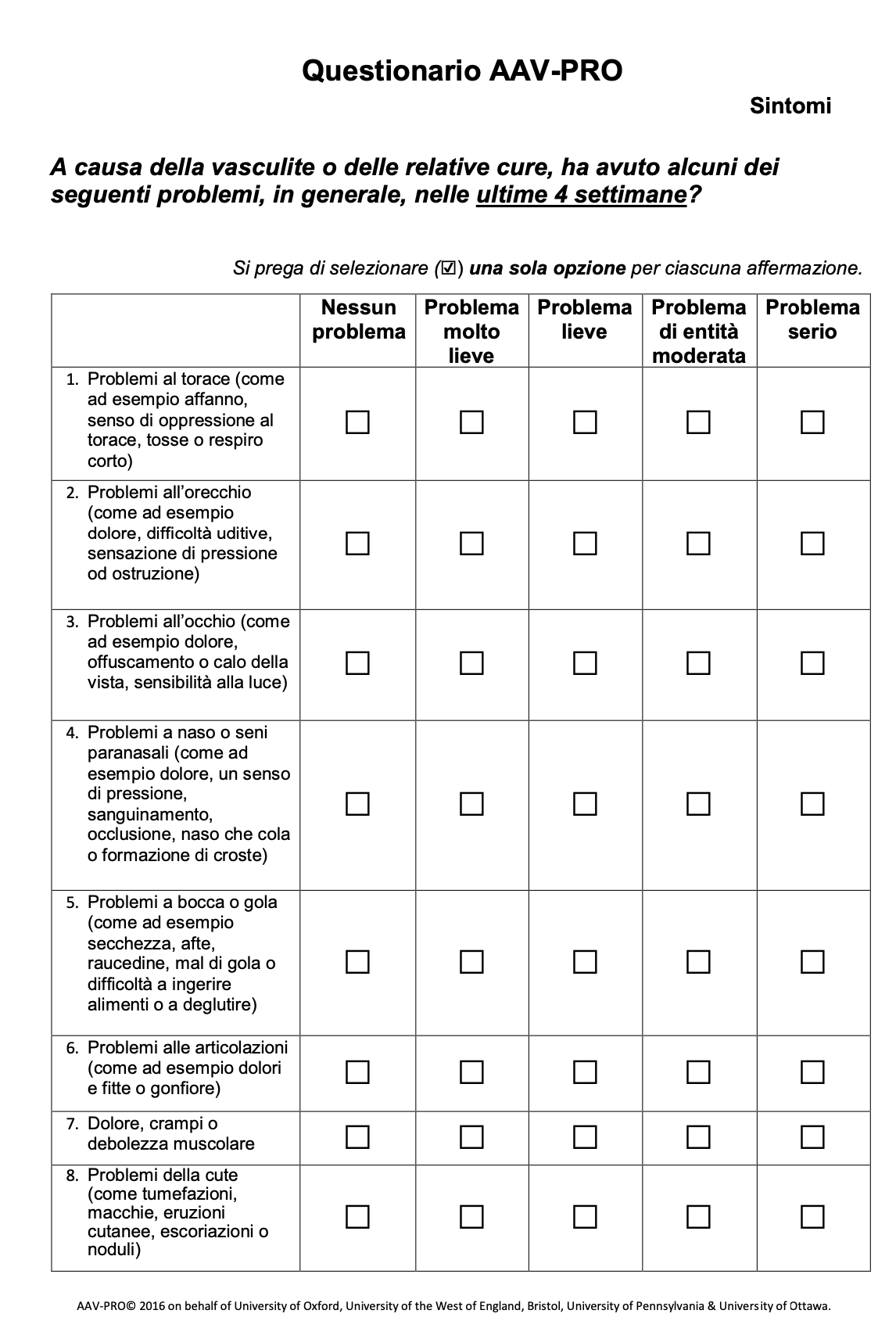

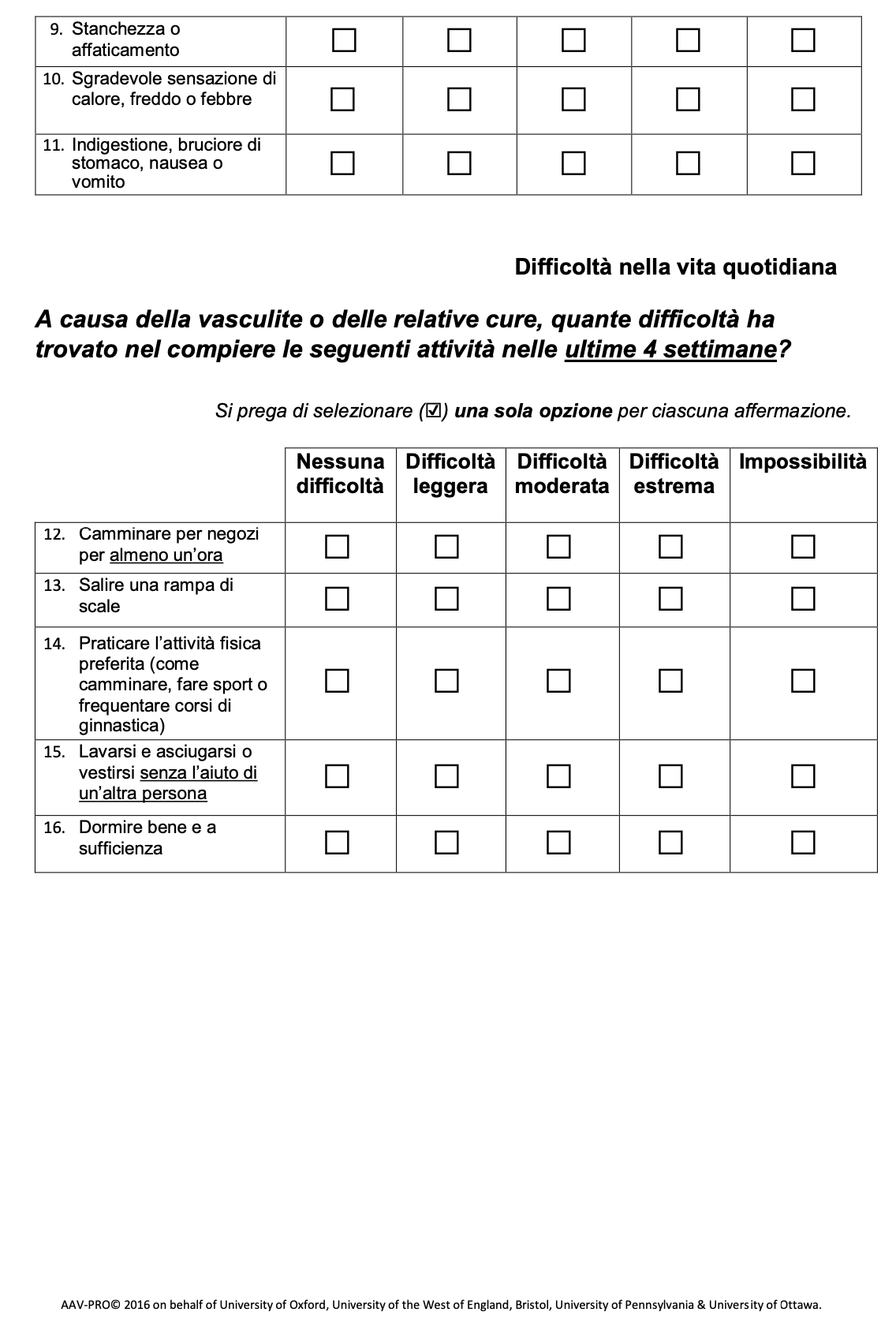

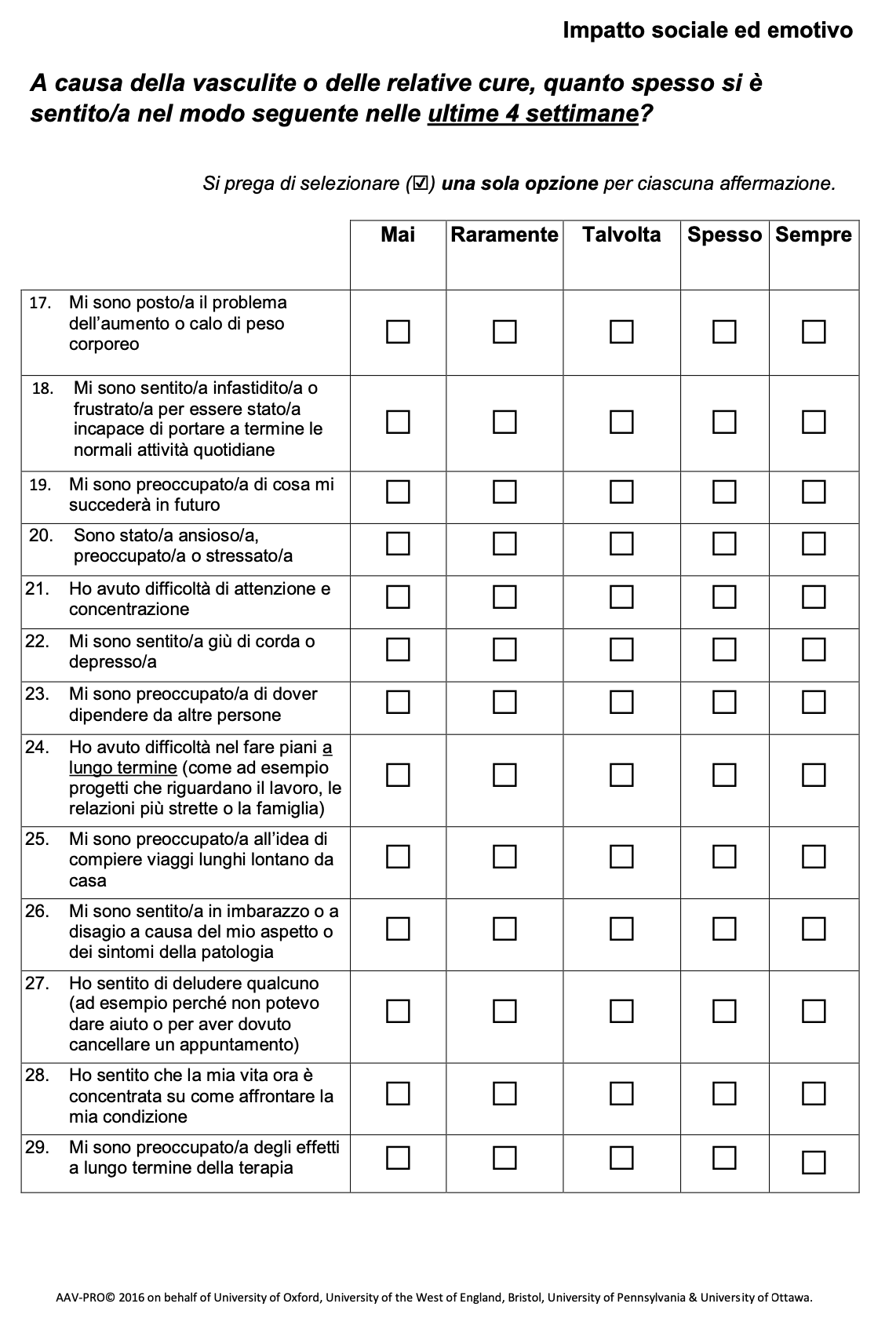

Supplement: rkae001_Supplementary_Data [file rkae001_supplementary_data.zip › 23-085 Supplementary Methods.docx]
